# Optimization of Parylene C and Parylene N thin films for use in cellular co-culture and tissue barrier models

**DOI:** 10.1038/s41598-023-31305-4

**Published:** 2023-03-14

**Authors:** Shayan Gholizadeh, Daniela M. Lincoln, Zahra Allahyari, Louis P. Widom, Robert N. Carter, Thomas R. Gaborski

**Affiliations:** 1 Department of Microsystems Engineering, Rochester Institute of Technology, 77 Lomb Memorial Drive, Rochester, NY 14623 USA; 2 Department of Biomedical Engineering, Rochester Institute of Technology, 160 Lomb Memorial Drive, Rochester, NY 14623 USA; 3 School of Chemistry and Materials Science, Rochester Institute of Technology, 84 Lomb Memorial Drive, Rochester, NY 14623 USA; 4 Department of Mechanical Engineering, Rochester Institute of Technology, 77 Lomb Memorial Drive, Rochester, NY 14623 USA

**Keywords:** Biomaterials - cells, Biomedical engineering

## Abstract

Parylene has been used widely used as a coating on medical devices. It has also been used to fabricate thin films and porous membranes upon which to grow cells. Porous membranes are integral components of in vitro tissue barrier and co-culture models, and their interaction with cells and tissues affects the performance and physiological relevance of these model systems. Parylene C and Parylene N are two biocompatible Parylene variants with potential for use in these models, but their effect on cellular behavior is not as well understood as more commonly used cell culture substrates, such as tissue culture treated polystyrene and glass. Here, we use a simple approach for benchtop oxygen plasma treatment and investigate the changes in cell spreading and extracellular matrix deposition as well as the physical and chemical changes in material surface properties. Our results support and build on previous findings of positive effects of plasma treatment on Parylene biocompatibility while showing a more pronounced improvement for Parylene C compared to Parylene N. We measured relatively minor changes in surface roughness following plasma treatments, but significant changes in oxygen concentration at the surface persisted for 7 days and was likely the dominant factor in improving cellular behavior. Overall, this study offers facile and relatively low-cost plasma treatment protocols that provide persistent improvements in cell-substrate interactions on Parylene that match and exceed tissue culture polystyrene.

## Introduction

There is an increasing interest in developing tissue-on-a-chip and barrier models, also known as microphysiological systems, due to their applications in drug discoveries and therapeutic strategies^1–4^. The blood–brain barrier (BBB) is highly studied among barrier models^5^. In vivo, the BBB limits drug delivery to the brain, and its dysfunction has been shown to play a major role in the onset of neurodegenerative diseases^6,7^. Given the prevalence of neurodegenerative diseases such as Alzheimer’s disease, the necessity for effective brain drug delivery, and limited treatment options, platforms such as physiologically representative in vitro BBB models are highly sought for disease modeling and drug discovery^8–10^. Porous membranes are integral components of these in vitro models^2,8,11–13^.

In barrier models, porous membranes serve as interfaces to establish a compartmentalized co-culture system between endothelial cells and another relevant cell type^14–20^. The two cell types are often grown on different sides of the membrane, and membrane pores facilitate paracrine signaling, direct physical contact, and even transmigration in some cases^16,21^. Polymeric track-etched membranes are usually embedded in versatile culture inserts such as Transwell® systems, and they are available with various pore sizes and porosities^2,16^. These are the most common membranes used for vascular barrier modeling^2^. However, these membranes have significant deficiencies, including limited live-imaging ability, irregular pore geometries, limited porosity, and, most importantly, thicknesses ranging from 5 to 10 µm (as opposed to the sub-micron endothelial-glial spacing observed in vivo) that significantly hinder cell–cell contact^16^. These shortcomings motivated the development of ultrathin nanomembrane technologies^2,16,22^.

Ultrathin nanomembranes often allow live imaging thanks to their optical transparency, effective cell–cell crosstalk due to thinness, and have higher porosities than their conventional membrane counterparts^16,21,22^. Despite these advances, almost all ultrathin and conventional membranes are made of synthetic materials with physical, chemical, and biological properties different from their biological analogs^2,13,15^. Remedies that compensate for this difference include membrane preincubation with cell culture media, extracellular matrix (ECM) coating, and plasma treatment^2,14,16,21,22^. Plasma treatment can be a more reproducible and effective method compared to other techniques to improve the biocompatibility of porous membranes^23–25^. However, it also encompasses complications such as transient effects and hydrophobic surface recovery and should be further investigated for different materials^16,26,27^.

It has been shown that plasma treatment affects organic and inorganic materials by enriching the surface with new oxygen-containing groups^28–31^. These effects fade relatively quickly, limiting the timeframe between membrane treatment and cell culture and creating inconsistent results due to time variability^32–34^. However, plasma treatment also creates surface roughness and micro- and nanotopographies as well as permanent changes in surface chemistry on organic materials such as synthetic polymers, which have been shown to improve cell attachment and biocompatibility through improving cell-substrate interactions^16,35,36^. Since well-attached cells deposit and assemble their own extracellular matrix (ECM) on the substrate, this can help mimic an in vivo microenvironment and be advantageous in creating a more physiologically representative barrier^8,37,38^. However, plasma treatment should be further explored and better understood for organic membrane material candidates in terms of physical and biological consequences. Parylene is among these candidate materials, and it has been previously examined for tissue barrier modeling^16,17,39^.

Parylene is a biocompatible synthetic polymer, often used to coat or seal Food and Drug Administration (FDA) approved or cleared devices, including medical implants in the human body^40,41^. Parylene has been successfully used for ultrathin membrane fabrication by our group as well as others, and it has generally shown favorable cell attachment and growth characteristics, especially after plasma treatment^16–18,42^. As part of our previous study, we briefly investigated whether the positive effects of plasma diminished over time by shelving the membranes for 7 or 14 days after treatment before use. Interestingly, cell attachment remained high even after 14 days of storage^16^. However, a deeper understanding of the plasma effects is needed to achieve an ideal membrane for barrier models, and the effects of induced roughness and surface chemistry should be differentiated.

In this study, we investigated the effects of oxygen plasma treatment on two commonly used biocompatible Parylene variants, Parylene C and Parylene N, and compared their results with tissue culture polystyrene as well as silicon dioxide. We switched from a semiconductor cleanroom reactive ion etching (RIE) tool used in our previous study to a more accessible benchtop plasma machine for facilitating future potential studies derived from this work. Additionally, we sought to focus on the effects and physical and chemical mechanisms of oxygen plasma treatment on Parylene. We opted to use non-porous, Parylene thin films to eliminate any effects of pores, pore edges, and porosity in how we interpret the effects of plasma treatment on cell-substrate interactions. We assessed two common endothelial cell types used for in vitro vascular barrier models to investigate the implications of varied plasma treatment lengths on cell-substrate interactions. We then characterized the Parylene surface in terms of hydrophobicity, physical roughness, and oxygen species content.

## Methods

### Membrane preparation

Thin film Parylene membrane fabrication was conducted using a protocol previously described^16^. Briefly, Micro-90, a water-soluble detergent, was deposited as a sacrificial layer by spin-coating on 6-inch silicon (100) wafers. We used Micro-90 on the silicon wafer to facilitate a simple Parylene film release from the wafer with water. 5 ml Micro-90 is spin-coated with a speed of 3000 rpm for 30 s. Parylene-C and Parylene N coating with the thicknesses of 500 nm was performed using DPX-C and DPX-N dimers (Specialty Coating Systems, USA), respectively, in an SCS Labcoter 2 Parylene deposition system (PDS 2010, Specialty Coating Systems, USA). We have previously confirmed 500 nm is the thinnest layer that we can reliably deposit without pinhole defects. Since the Parylene films used in this study were not released as free-standing membranes, potential pinhole defects were not of concern. The process began at a base chamber pressure of 10 mTorr, and the dimer-cracking furnace was heated to 690 °C for Parylene C and 650 °C for Parylene N. Then, the vaporizer was ramped to a final temperature of 175 °C or 160 °C for Parylene C and Parylene N, respectively, causing the sublimation of the dimer. The temperature ramp rate of the vaporizer was controlled to maintain a chamber pressure of 25 mTorr.

### Oxygen plasma treatment

Unlike our previous studies, which used high-cost semiconductor cleanroom equipment with limited availability, such as inductively coupled plasma RIE tools, we aimed to use a benchtop plasma treatment tool that is more readily available. For this purpose, a Harrick PDC-001 plasma cleaner (Harrick, NY, USA) was used at its high-power setting for either 10 or 20 min, based on previous use of this tool for surface activation. This plasma treatment tool was used since it is a widely available and a relatively low-cost tool as opposed to the more expensive and complex reactive ion etching (RIE) tools. Samples were stored for 1–7 days after treatment to study the longevity of the plasma effects before use.

### 3D printing scaffolds and membrane assembly

A Form 3B 3D printer (Formlabs, MA, USA) was used to fabricate cell culture scaffolds capable of supporting cell culture in multi-well plates. Briefly, a 3D mechanical design software (SolidWorks, MA, USA) was used to design scaffolds fitting in 24-well plates. The scaffolds were fabricated using BioMed Amber Resin (Formlabs, MA, USA) with a resolution set to 50 µm. Smooth laser-cut polymethylmethacrylate (PMMA) circular parts were attached to double-sided pressure-sensitive adhesive (MP468, 3 M, MN, USA) on both sides and were pushed on pieces of membrane-coated silicon wafers. Parylene membranes attached to the 3D-printed scaffolds were released by dipping in deionized (DI) water from the silicon substrate.

### Cell culture

Pooled human umbilical vein endothelial cells (HUVECs) and a human cerebral microvascular endothelial cell line (hCMEC/D3) were cultured in EGM™-2 MV consisting of EBM™-2 and EGM™-2 MV SingleQuots™ Kit with 2.4% FBS, and 1% penicillin and streptomycin. HUVECs and cell culture reagents were purchased from Lonza (Walkersville, MD, United States). hCMEC/D3 cells were purchased from EMD Millipore (Temecula, CA, USA). HUVECs and hCMEC/D3 cells were detached by TrypLE and seeded on samples at a density of 2 × 10^4^ cells/cm^2^. Cells were used between passages 4 − 6.

### Cell spread area

Cells were seeded on the Parylene C and Parylene N samples for 24 h, fixed in 3.7% formaldehyde for 15 min, and washed with PBS. This was followed by cell permeabilization for 3 min in 0.1% Triton X-100 and washing with double-distilled water. To visualize nuclei and cytoskeletons, HUVECs were stained using DAPI (300 nM) and 1:400 Alexa Fluor 488 conjugated phalloidin for 3 and 15 min, respectively. Cells were washed with PBS. Images were captured at 20 × magnification through DAPI and GFP filters on a Keyence BZ-X700 microscope (Keyence Corp. of America, MA, USA). The area of spreading of actin fibers was measured for each cell using ImageJ software.

### Fibronectin fibrillogenesis

Fibronectin fibrillogenesis was evaluated on Parylene C and Parylene N samples. After cell culture for 24 h, cells were fixed with 3.7% formaldehyde for 15 min, washed with PBS, blocked with 20 mg/mL BSA for 15 min, and then washed again with PBS. Cells were stained using a 1:100 dilution of Alexa Fluor 488 conjugated anti-fibronectin (clone FN-3) for 2 h, and washed with PBS three times. PBS was added to the samples, and they were imaged through a GFP filter using a Keyence BZ-X700 microscope. The lengths of fibrils were measured using the CT-FIRE package^43^, which was developed by the Laboratory for Optical and Computational Instrumentation at the University of Wisconsin-Madison to extract individual collagen fibers from images automatically.

### Collagen IV deposition

Collagen IV deposition was also evaluated on Parylene C and Parylene N samples through immunofluorescence microscopy. Similar to the fibronectin fibrillogenesis assay, cells were fixed with 3.7% formaldehyde for 15 min, washed with PBS, blocked with 20 mg/mL BSA for 15 min, and then washed again with PBS. Samples were incubated with 1:100 diluted Fluor 647 conjugated anti-collagen IV (clone 1042) for 4 h and imaged through a Cy5 filter using a Keyence BZ-X700 microscope. Collagen IV coverage was quantified using the thresholding function of ImageJ software (National Institutes of Health, USA).

### Contact angle measurement

The hydrophilicity of the surface of Parylene C-coated glass coverslips was evaluated through static contact angle measurements. For each condition, three different samples were used. 10 µl of DI water was placed at the center of the samples, and images were taken. Edges were omitted as these are typically also avoided in all microfabricated samples for reliability. The mean contact angle was extrapolated from three measurements for each sample via ImageJ software.

### Atomic force microscope measurement

Parylene C and Parylene N membranes (untreated, treated for 10 min, and treated for 20 min) were examined using a MultiMode 8-HR atomic force microscope (AFM) (Bruker, USA). TR800PSA cantilevers (Oxford Instruments) with a spring constant of 0.15 N/m were utilized to scan the surface in tapping mode. The samples were prepared, mounted onto the AFM cell chamber, and surface topography scans were taken with a scan speed of 0.5 Hz to minimize imaging artifacts over a fixed area.

### X-ray photoelectron spectroscopy (XPS)

X-ray photoelectron spectroscopy (XPS) was used to evaluate the effects of plasma treatment on the surface chemistry (1–10 nm depth) and its longevity over 7 days. XPS spectra were collected using a Kratos Axis Ultra DLD XPS system (Kratos Analytical, UK). After chamber pump-down and surface contaminant removal, survey scans with 1 eV steps and high-resolution scans for oxygen and carbon with 0.1 eV steps were collected. Shirley background was used to correct collected spectra, and CasaXPS was utilized to deconvolute peaks and correct the spectra by shifting the carbon–carbon peak to 284.8 eV. The area under the curve for the oxygen peak was normalized to the corresponding area for aliphatic C, and the oxygen peak area was used as a normalized representation of oxygen surface concentration.

### Statistical analysis

Cell culture experiments were performed in triplicate (three independent devices) unless otherwise stated. Each experimental replicate was performed on separate devices. Experimental groups and controls were performed in parallel (one replicate for each group per day). Results are reported in mean ± standard error of the mean (SEM). Statistical analysis was performed in Prism (GraphPad Software, Inc., San Diego, CA). For statistical significance, *, **, *** and **** indicate p-values of less than 0.05, 0.01, 0.001 and 0.0001, respectively.

## Results and discussion

### Cell spreading

**Figure 1:**
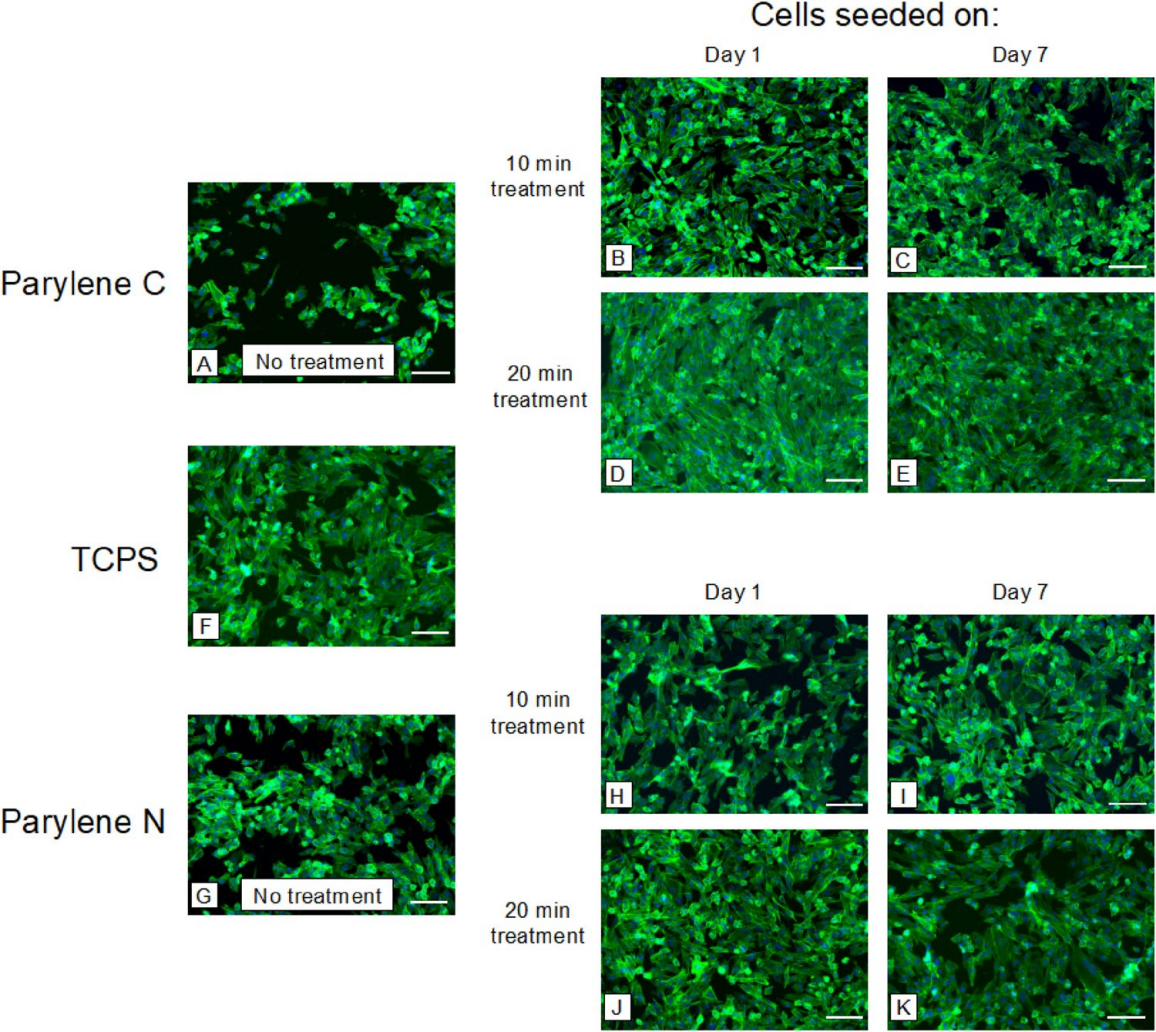
Spreading of vascular endothelial cells (HUVECs) on plasma-treated and untreated samples. Samples were treated for either 10 or 20 min of oxygen plasma, and cells were seeded on the substrates on day 1 or day 7 to investigate the effect of plasma treatment and its persistence. Representative images of nuclei (DAPI, blue) and F-actin (phalloidin, green) after 24 h on (**A–E**) Parylene C membranes, (**F**) TCPS, and (**G–K**) Parylene N membranes. Scale bar = 100 μm in all images.

**Figure 2:**
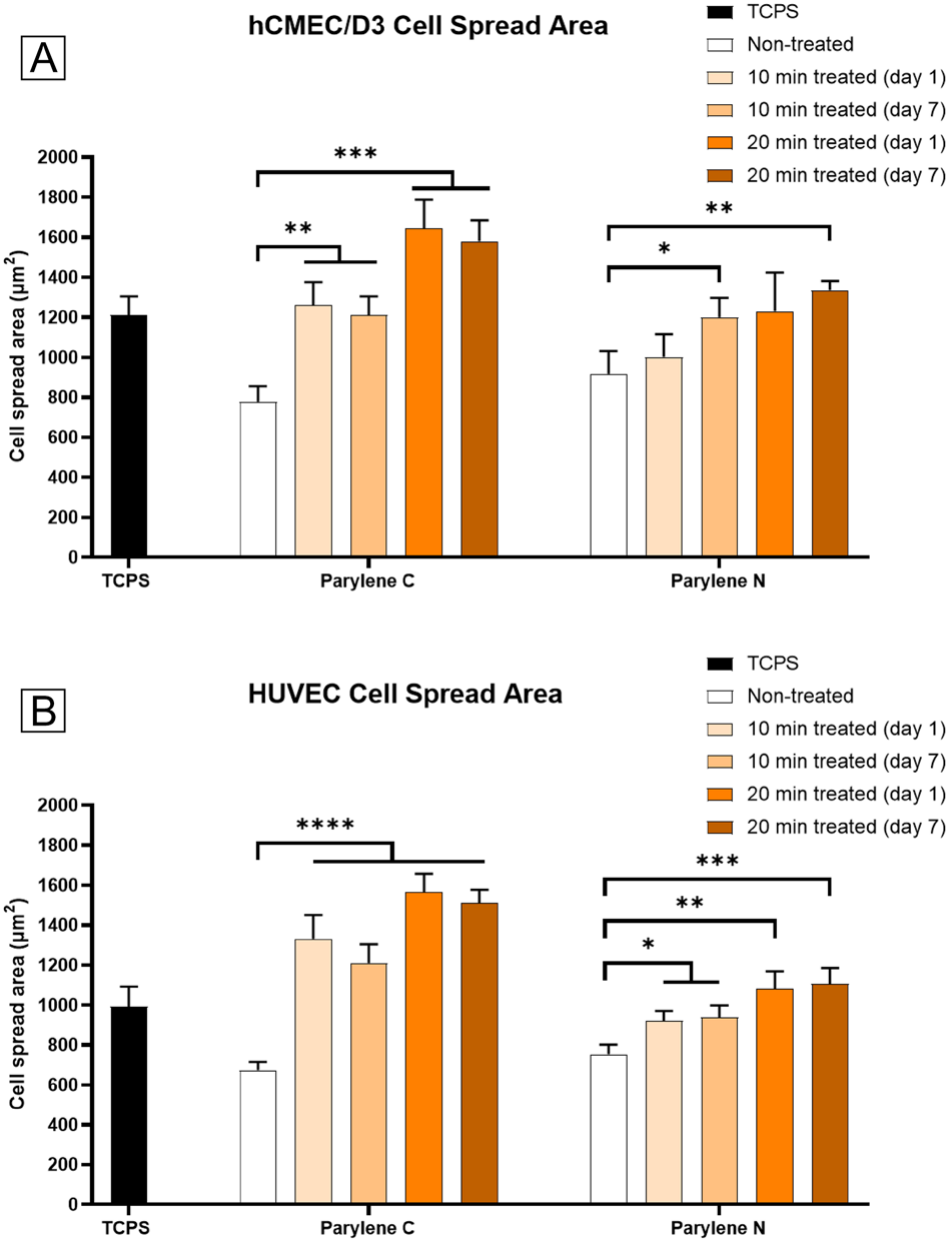
Changes in cell spread area of vascular endothelial cells on Parylene C and Parylene N membranes. (**A**) hCMEC/D3 cell spread area on TCPS, Parylene C, and Parylene N membranes. (**B**) HUVEC cell spread area on TCPS, Parylene C, and Parylene N membranes.

Two endothelial cell types commonly used for vascular barrier studies (hCMEC/D3 and HUVEC) were used for cell-substrate interaction experiments. Instead of cell viability, we aimed to study cell spread area as a more direct measure of cell-substrate interaction. Our results demonstrated that plasma treatment increased the cell spread area for both treatment times, while more extended treatment led to a marginally higher spread area for HUVECs (Fig. 1). Oxygen plasma treatment led to more cell spreading in Parylene C, even outperforming the spreading on TCPS. However, oxygen plasma treatment of Parylene N led to less pronounced improvement and yielded spreading within the same levels of spreading on TCPS. This becomes more evident in the subsequent analysis (Fig. 2).

Figure 2 demonstrates cell spread analysis of HUVECs and hCMEC/D3 cells on Parylene C and Parylene N with different treatment and test conditions. It can be noted that although untreated Parylene C showed the smallest cell spread area, treated Parylene C membranes showed the highest cell spread area as compared to Parylene N membranes and TCPS. Another critical finding was that even though there was a slight effect of hydrophobic recovery in treated Parylene samples, there was no decrease in cell spread area on day 7 samples as compared to those samples seeded on day 1 after treatment, possibly due to permanent changes in surface properties.

Another important observation is that there was no statistically significant improvement with 20 min treatment as compared to 10 min treatment except for hCMEC/D3s on Parylene C. This likely points to a saturation-like event either in surface roughness or surface bonds which will be explored in material characterizations in the following sections.

### Fibronectin fibrillogenesis

**Figure 3:**
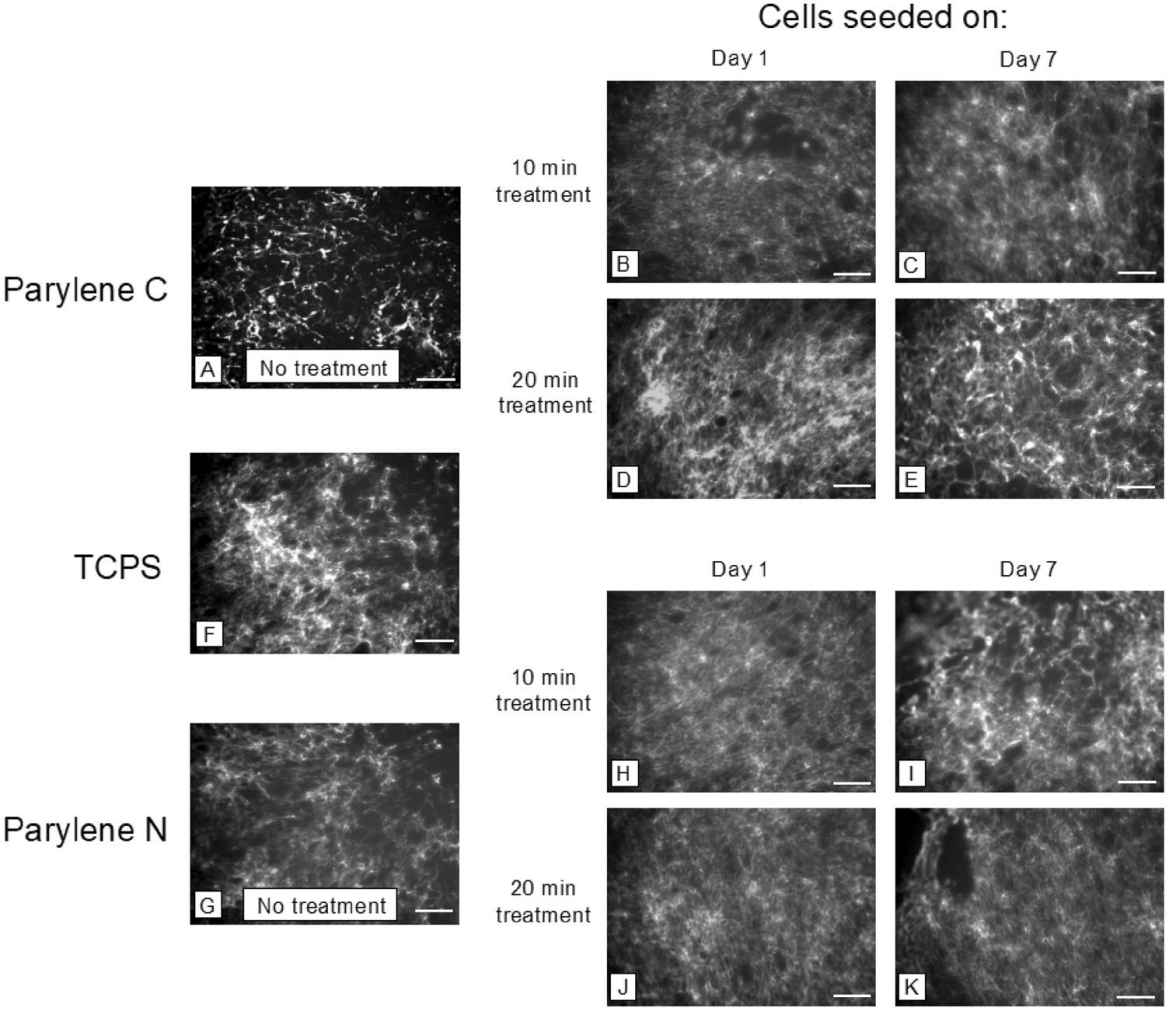
Representative images of fibronectin fibrillogenesis by vascular endothelial cells on (**A–E**) Parylene C membranes, (**F**) TCPS, and (**G–K**) Parylene N membranes. Scale bar = 100 μm in all images.

**Figure 4:**
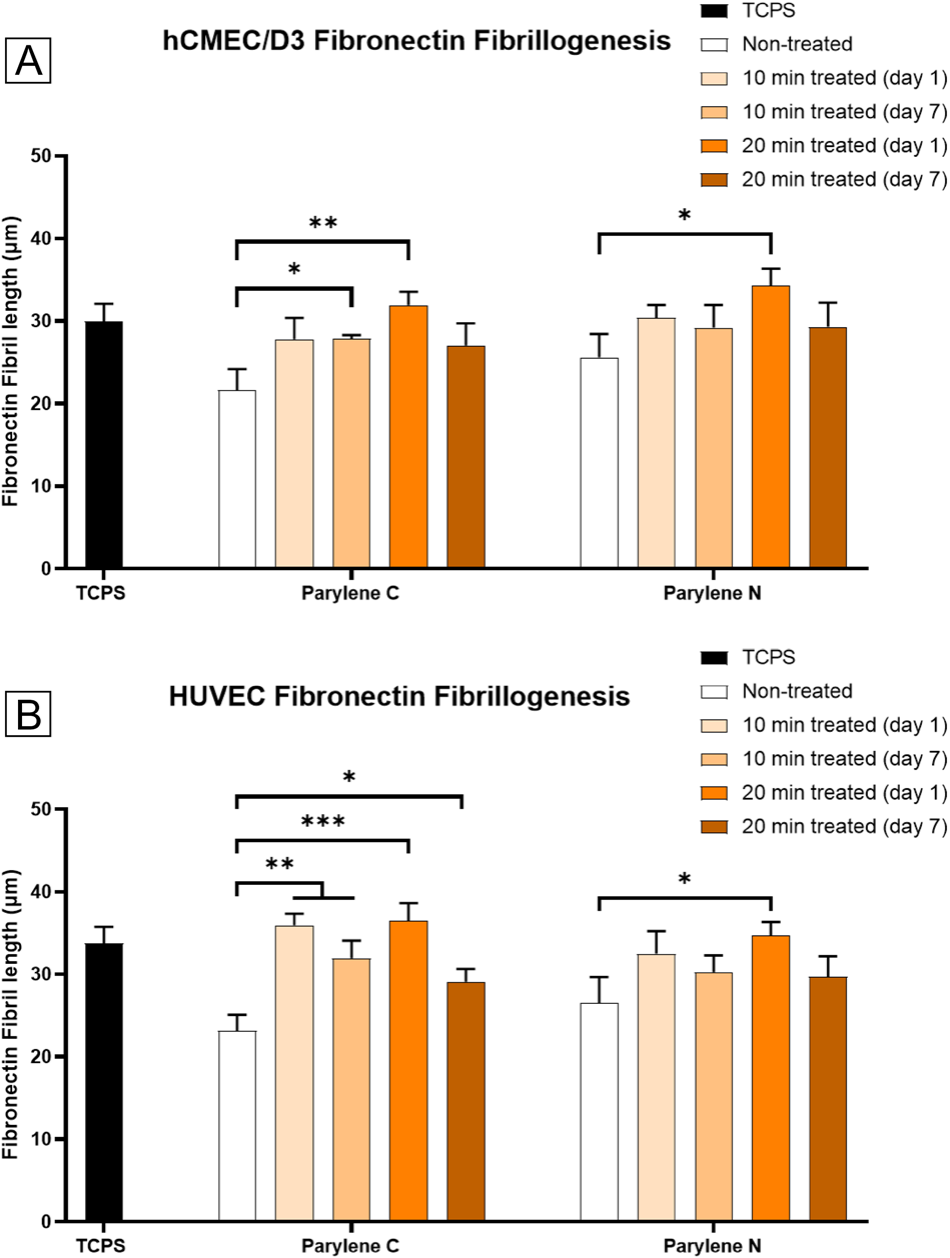
Changes in fibronectin fibrillogenesis by vascular endothelial cells on Parylene C and Parylene N membranes result from plasma treatment. (**A**) hCMEC/D3 fibronectin fibril lengths on TPCS, Parylene C, and Parylene N membranes. (**B**) HUVEC fibronectin fibril lengths on TCPS, Parylene C, and Parylene N membranes.

Fibronectin fibrillogenesis is associated with a cell’s ability to generate high traction forces^44–47^. Disruption in fibronectin fibrillogenesis can indicate lesser cell-substrate interaction and lower integrity in the formed endothelial barrier. Our results on fibronectin fibrillogenesis on hCMEC/D3 cells and HUVECs indicated that treatment of Parylene C and Parylene N led to a similar improvement in fibronectin fibrillogenesis (Figs. 3, 4).

However, there is a noticeable decrease in fibronectin fibrillogenesis in most cases from day 1 to day 7, indicating that even with the perceived role of surface roughness, there is a somewhat apparent deterioration in the activated substrate. This decrease is only statistically significant in 20 min treated samples, indicating a persistent surface activation baseline. Despite this decrease, the results in all treatment conditions were still comparable to TCPS on day 7.

Another important observation is that only some of the day 1 samples were able to outperform TCPS in terms of fibrillogenesis. This contrasts cell spreading, where many of the treated Parylene samples demonstrated larger cell areas. All 16 sets showed statistically significant fibrillogenesis enhancement over untreated samples, cementing the persistent nature of Parylene plasma activation.

### Collagen IV protein expression

The interaction of endothelial β1-integrins with collagen IV of the basement membrane is correlated to the expression of the tight junction protein claudin-5 and barrier integrity in vitro^48^. The interaction through the integrin receptors provides physical support and regulates signaling pathways, whereby the endothelial cells can adapt to changes in the microenvironment. Therefore, the ability of endothelial cells to deposit collagen IV can be vital to having more physiologically representative in vitro barrier models.

**Figure 5:**
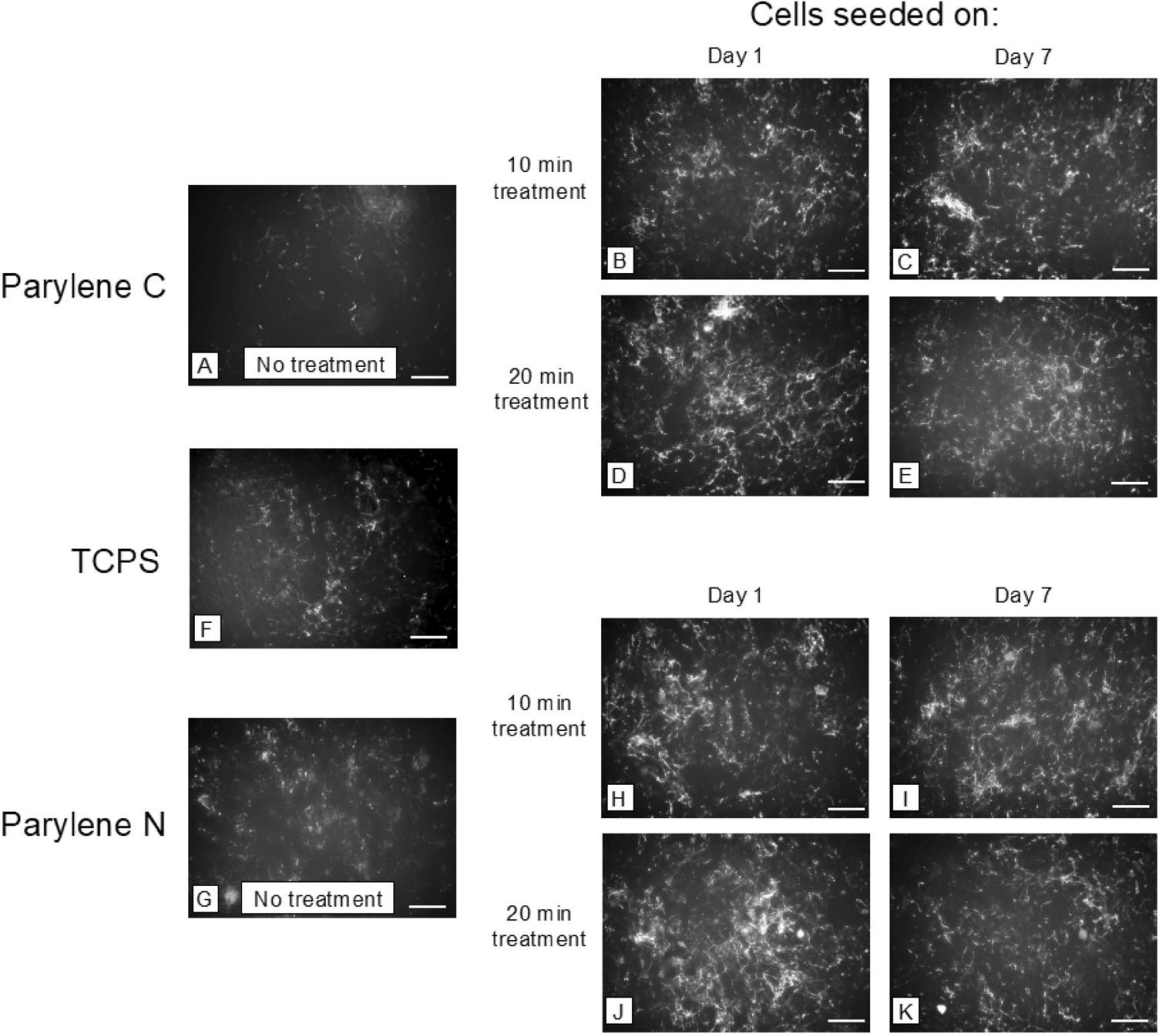
Representative images of Collagen IV deposition by vascular endothelial cells (HUVECs) on (**A–E**) Parylene C membranes, (**F**) TCPS, and (**G–K**) Parylene N membranes. Scale bar = 100 μm in all images.

**Figure 6:**
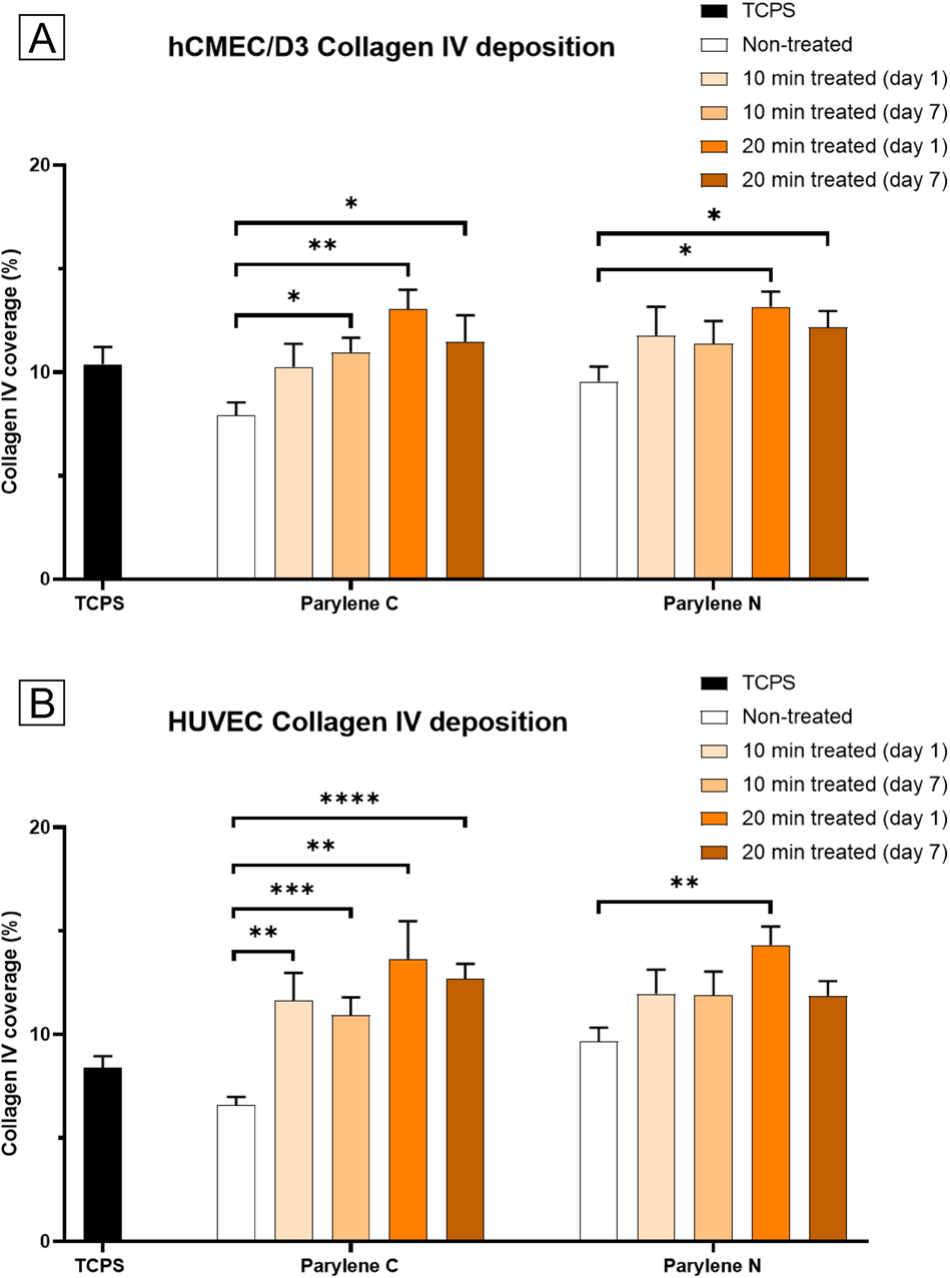
Changes in collagen IV deposition by vascular endothelial cells on Parylene C and Parylene N membranes result from plasma treatment. (**A**) hCMEC/D3 collagen IV coverage on TCPS, Parylene C, and Parylene N membranes. (**B**) HUVEC collagen IV coverage on TCPS, Parylene C, and Parylene N membranes.

Our results display significant improvement in collagen IV deposition due to plasma treatment across all samples, and they either perform comparably or better than TCPS (Figs. 5, 6). The decrease from day 1 to day 7 is still observed in 20 min treated samples but is much less pronounced than for fibronectin fibrillogenesis. The results are promising for in vitro barrier models in terms of reliability, persistency, and effectiveness of plasma treatment for collagen IV deposition.

### Hydrophilicity and hydrophobic recovery

**Figure 7:**
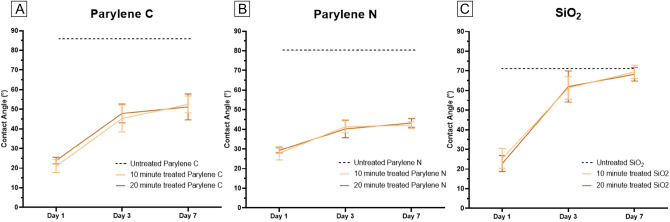
Contact angle measurements from day 1, day 3, and day 7 of plasma treatment for (**A**) Parylene C, (**B**) Parylene N, and (**C**) SiO_2_ surfaces. The dashed line in each plot represents the measurements from the respective untreated material.

It is well-known that hydrophilic surfaces promote cell adhesion. Previous work on the underlying mechanisms of plasma-induced surface activation has mainly focused on surface energy and surface chemistry with valuable insights and outcomes^49,50^. Silicon dioxide (SiO_2_) thin films and coatings are commonly used in bioMEMS as cell culture and biosensor substrates. The water contact angle of SiO_2_ is well known to respond strongly to oxygen plasma, becoming very hydrophilic, but with a relatively rapid hydrophobic recovery over time. We measured the contact angle and hydrophobic recovery of Parylene C and N and compared them to SiO_2_. Day 1 (1 h after treatment), day 3, and day 7 measurements following plasma treatment show that Parylene C and Parylene N both exhibited much lower contact angles and higher hydrophilicity compared to untreated samples, and they retained this characteristic with only moderate hydrophobic recovery (Fig. 7). SiO_2_ thin films, however, showed almost complete recovery by day 7, as expected. All samples were treated on the same day and exposed to the same ambient conditions over 7 days. These data suggest the plasma treatment of Parylene resulted in a more permanent surface property change, possibly due to an increase in roughness leading to higher surface charge density, helping maintain hydrophilicity even after 7 days.

### Surface morphological evaluation

**Figure 8:**
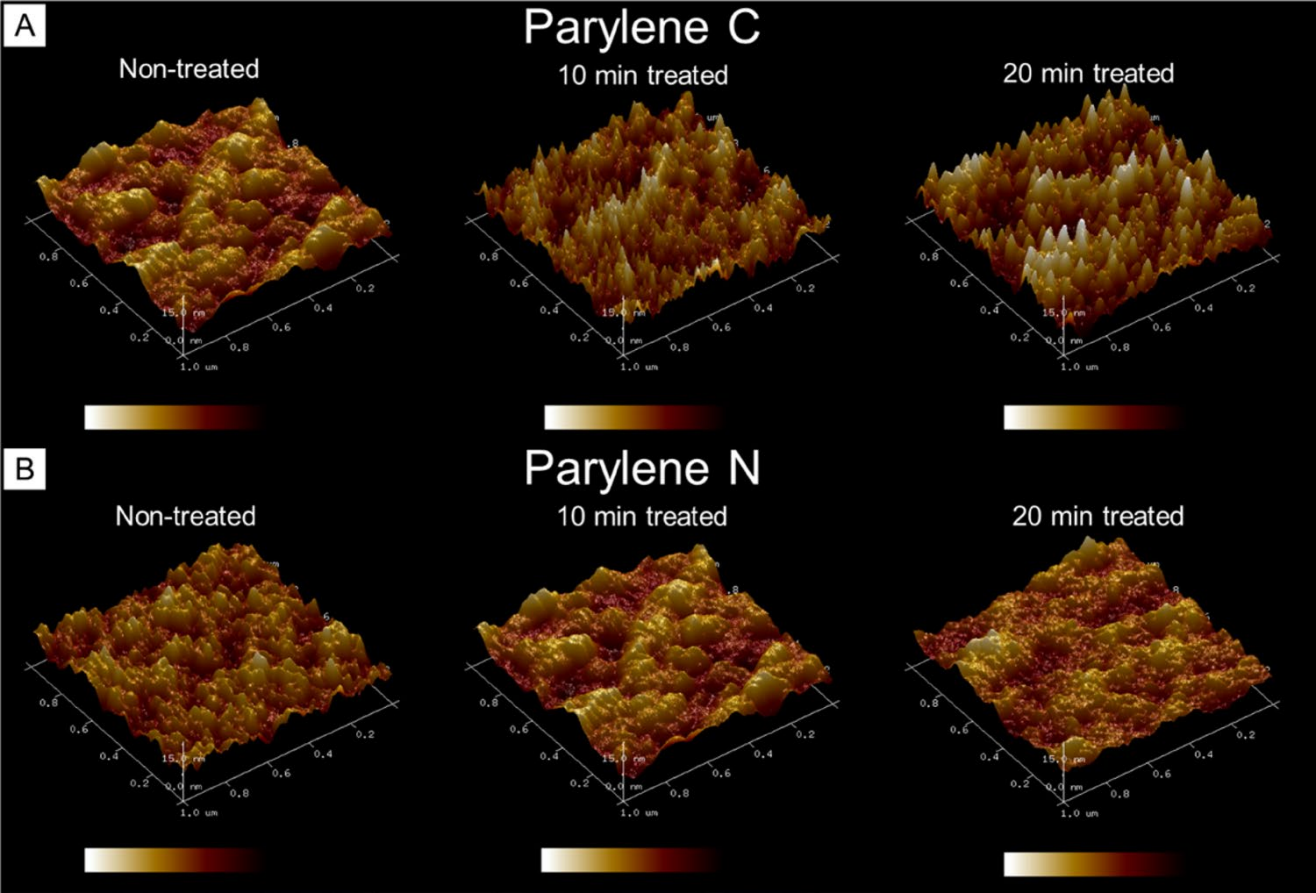
The effect of surface plasma modification of different lengths of time on surface topography and roughness. AFM images of Parylene C and Parylene N surfaces demonstrated slight increases in introduced surface area and roughness on plasma-treated Parylene C as the treatment time increased. Noticeable changes were not observed in plasma-treated Parylene N. The X–Y (0–1 µm) and Z-axes (0–15 nm), as well as the intensity mapping, are all set to the same scale in each AFM image.

In previously published works, it was observed that plasma activation had shown persistency over time for some organic surfaces^16,51,52^. Surface morphological or roughness changes could explain why some polymeric materials exhibit slower hydrophobic recovery and more persistent effects. Parylene C and Parylene N have minor chemical structure differences, but AFM measurements demonstrate distinct differences in surface roughness between the two variants (Fig. 8). Parylene C shows a slight increase in roughness as plasma treatment time increased, but minimal, if any changes are noted in Parylene N. The changes in roughness are minimal, and considering the differences between Parylene C and N in Fig. 8, enhanced roughness is not likely the mechanism of improved cell-substrate interactions on these materials following plasma treatment.

### X-ray photoelectron spectroscopy (XPS)

**Figure 9:**
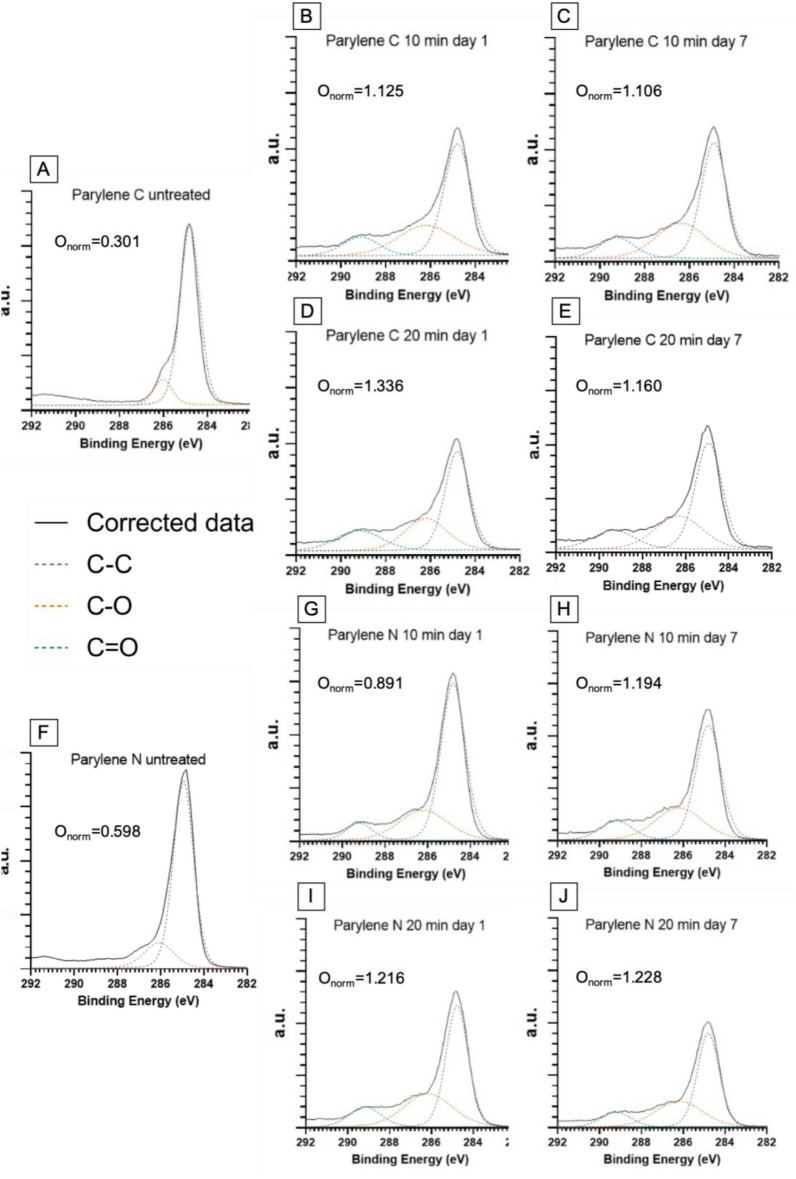
XPS visualization of high-energy tails associated with carbon bonding to oxygen for Parylene C (**A-E**) and Parylene N (**F-J**) membranes with different treatments on day 1 and day 7 and corresponding normalized oxygen concentrations extracted from the survey scans. Continuous black lines in each graph correspond to the corrected data, and the dotted lines are Gaussian–Lorentzian fits for carbon–oxygen (orange and blue) and carbon–carbon (gray) bonds.

As opposed to the surface topography analysis with AFM, where only mild increases in Parylene C roughness were observed, XPS spectra analysis showed significant changes after 20 min of plasma treatment that persisted at 7 days for both Parylene C and N (Fig. 9). Previous studies established a positive correlation between the presence of oxygen-containing surface groups and cell biocompatibility was established^30,53–58^. More specifically, it was shown that oxygen-containing groups can increase hydrophilicity and a cell’s ability to adhere and spread on the substrate and deposit more organized extracellular matrix proteins^58,59^. We found that plasma treatment increased the surface oxygen bonds (C–O and C=O), with the longer 20 min treatment leading to the highest amount of oxygen species. This mirrored the observed changes in cell interactions on the treated Parylene substrates. Similar to the increase in endothelial cell spreading on Parylene C compared to Parylene N after plasma treatment, the increase in oxygen species in Parylene C was also greater. This suggests that changes in surface chemistry likely govern the cell-substrate interactions on Parylene C and Parylene N substrates and play a more significant role than surface topography.

## Conclusion

In this study, we investigated oxygen plasma treatment of Parylene C and N with the goal of improving and understanding cell-substrate interactions of these materials that can be used in cellular co-culture and tissue barrier models. In general, we found that plasma treatment improved the spreading of vascular endothelial cells as well as increased the production of extracellular matrix and basement membrane proteins to levels equivalent to or higher than tissue culture polystyrene. We investigated two different treatment times as well the persistence of effects over 7 days. We found that physical and chemical changes persisted in Parylene substrates, with the greatest effects in Parylene C. Surprisingly, we found relatively modest changes in physical roughness that likely could not alone explain improved cell-substrate interactions. Instead, we found significant changes in oxygen species in both Parylene C and N that persisted for an entire week, consistent with relatively low hydrophobic recovery compared to substrates such as SiO_2_. In summary, we show that a low-cost benchtop oxygen plasma chamber can modify Parylene C to produce a cell-friendly culture substrate with similar properties to tissue culture polystyrene.

## Data Availability

The datasets used and analyzed during the current study are available from the corresponding author upon reasonable request.
