# Publisher Correction: Optimization of Parylene C and Parylene N thin films for use in cellular co-culture and tissue barrier models

**DOI:** 10.1038/s41598-023-32725-y

**Published:** 2023-04-07

**Authors:** Shayan Gholizadeh, Daniela M. Lincoln, Zahra Allahyari, Louis P. Widom, Robert N. Carter, Thomas R. Gaborski

**Affiliations:** 1 Department of Microsystems Engineering, Rochester Institute of Technology, 77 Lomb Memorial Drive, Rochester, NY 14623 USA; 2 Department of Biomedical Engineering, Rochester Institute of Technology, 160 Lomb Memorial Drive, Rochester, NY 14623 USA; 3 School of Chemistry and Materials Science, Rochester Institute of Technology, 84 Lomb Memorial Drive, Rochester, NY 14623 USA; 4 Department of Mechanical Engineering, Rochester Institute of Technology, 77 Lomb Memorial Drive, Rochester, NY 14623 USA

Correction to: *Scientific Reports* 10.1038/s41598-023-31305-4, published online 14 March 2023

The original version of this Article contained an error in Figure 9, where the O_norm_ data in panels (A)–(J) did not display correctly.

The original Figure 9 and accompanying legend appear below.

**Figure 9:**
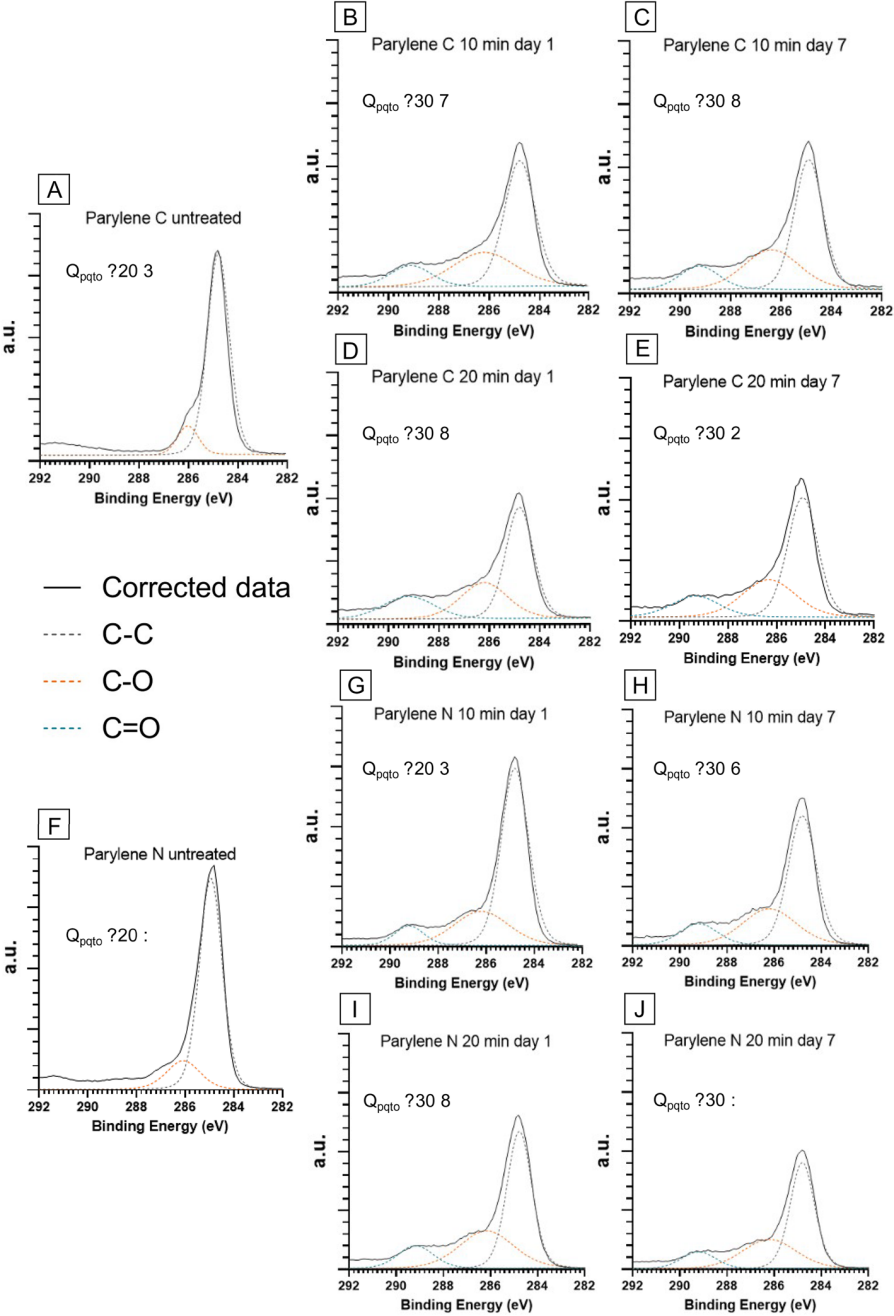
XPS visualization of high-energy tails associated with carbon bonding to oxygen for Parylene C (**A–E**) and Parylene N (**F–J**) membranes with different treatments on day 1 and day 7 and corresponding normalized oxygen concentrations extracted from the survey scans. Continuous black lines in each graph correspond to the corrected data, and the dotted lines are Gaussian–Lorentzian fits for carbon–oxygen (orange and blue) and carbon–carbon (gray) bonds.

The original Article has been corrected.

